# Systematic evaluation of the pre-eclampsia drugs, dietary supplements and biologicals pipeline using target product profiles

**DOI:** 10.1186/s12916-022-02582-z

**Published:** 2022-11-04

**Authors:** Annie R. A. McDougall, Roxanne Hastie, Maya Goldstein, Andrew Tuttle, Stephen Tong, Anne Ammerdorffer, A. Metin Gülmezoglu, Joshua P. Vogel

**Affiliations:** 1 Maternal, Child and Adolescent Health Program, Burnet Institute, 85 Commercial Road, Melbourne, VIC 3004 Australia; 2 Department of Obstetrics and Gynaecology, University of Melbourne, Heidelberg, Australia; 3 Policy Cures Research, Sydney, Australia; 4 Concept Foundation, Geneva, Switzerland; 5 School of Public Health and Preventive Medicine, Monash University, Melbourne, Australia

**Keywords:** Drug development, Vitamin D, Esomeprazole, l-Arginine, Metformin, Sulfasalazine, Chloroquine, Maternal medicine, Pregnancy, Hypertension

## Abstract

**Background:**

The Accelerating Innovation for Mothers (AIM) project established a database of candidate medicines in research and development (R&D) between 2000 and 2021 for five pregnancy-related conditions, including pre-eclampsia. In parallel, we published target product profiles (TPPs) that describe optimal characteristics of medicines for use in preventing/treating pre-eclampsia. The study objective was to use systematic double screening and extraction to identify all candidate medicines being investigated for pre-eclampsia prevention/treatment and rank their potential based on the TPPs.

**Methods:**

Adis Insight, Pharmaprojects, WHO international clinical trials registry platform (ICTRP), PubMed and grant databases were searched (Jan–May 2021). The AIM database was screened for all candidates being investigated for pre-eclampsia. Candidates in clinical development were evaluated against nine prespecified criteria from TPPs identified as key for wide-scale implementation, and classified as high, medium or low potential based on matching to the TPPs. Preclinical candidates were categorised by product type, archetype and medicine subclass.

**Results:**

The AIM database identified 153 candidates for pre-eclampsia. Of the 87 candidates in clinical development, seven were classified as high potential (*prevention*: esomeprazole, l-arginine, chloroquine, vitamin D and metformin; *treatment*: sulfasalazine and metformin) and eight as medium potential (*prevention*: probiotic lactobacilli, dalteparin, selenium and omega-3 fatty acid; *treatment*: sulforaphane, pravastatin, rosuvastatin and vitamin B3). Sixty-six candidates were in preclinical development, the most common being amino acid/peptides, siRNA-based medicines and polyphenols.

**Conclusions:**

This is a novel, evidence-informed approach to identifying promising candidates for pre-eclampsia prevention and treatment — a vital step in stimulating R&D of new medicines for pre-eclampsia suitable for real-world implementation.

**Supplementary Information:**

The online version contains supplementary material available at 10.1186/s12916-022-02582-z.

## Background

Pre-eclampsia and eclampsia, the most common hypertensive disorders of pregnancy, are a leading cause of maternal mortality, affecting approximately 4.6% of pregnant women [1] and resulting in approximately 14% of maternal deaths worldwide [2, 3]. Women in low- and middle-income countries (LMICs) carry most of this burden; a quarter of maternal deaths in Latin America and a tenth of maternal deaths in Asia and Africa are due to pre-eclampsia/eclampsia [4]. Pre-eclampsia results from abnormal placental development associated with a pro-inflammatory response and an imbalance of angiogenic factors [5].

There are few effective medicines for the prevention and treatment of pre-eclampsia. The World Health Organization (WHO) recommends that women at increased risk of pre-eclampsia (such as women with diabetes, chronic hypertension or a previous history of pre-eclampsia) receive prophylaxis with low-dose aspirin initiated before 16 weeks’ gestation [6]. Additionally, daily calcium supplementation can be offered to women living in regions with low calcium intake to reduce their likelihood of developing pre-eclampsia [7]. For women who develop severe pre-eclampsia, magnesium sulphate is recommended to prevent or treat eclamptic seizures [4]. None of the medicines currently recommended address the underlying aetiology for this complex condition.

A significant barrier to reducing the global burden of pre-eclampsia is the “drug drought” — a lack of medicines for pregnancy-specific conditions [8, 9]. Despite an estimated 295,000 maternal deaths globally each year [10], governments and pharmaceutical companies have largely not invested in maternal medicine development, driven in part by the enhanced liability risks of clinical research involving pregnant women and a perceived small target population [11, 12]. The Accelerating Innovation for Mothers (AIM) project was initiated to address these structural issues and to play a catalytic role in the development of new medicines for obstetric conditions [13].

Through a multi-stage, mixed methods study, we developed two target product profiles (TPPs) for new medicines to prevent and treat pre-eclampsia [14]. TPPs are strategic documents that identify upfront the key characteristics that a new product should demonstrate in order to meet a specific clinical or public health need [15]. TPPs have been used to drive research and development (R&D) in vaccines, diagnostics and therapeutics for multiple diseases and conditions [16–19]. In the current study, we aimed to analyse the AIM database of the historic and current pipeline of drugs, biologics and dietary supplements for pre-eclampsia and evaluate them against the TPPs, to identify those candidates that are most promising in terms of reducing pre-eclampsia-related morbidity and mortality globally.

## Methods

### AIM database of the drug development pipeline

Development of the AIM database has been previously described [20, 21]. Briefly, the database was populated by searching Adis Insight, Pharmaprojects, WHO international clinical trials registry platform (ICTRP), PubMed and grant databases to identify candidate medicines investigated for five priority maternal conditions (pre-eclampsia, preterm birth/labour, postpartum haemorrhage, foetal growth restriction and foetal distress). The AIM project database identified a total of 444 candidates for the period Jan 2000 to May 2021. For pre-eclampsia/eclampsia, 153 candidates were identified.

To identify higher-potential candidates, we applied a systematic, stepwise approach to all 153 candidates for pre-eclampsia prevention and treatment. First, we excluded candidates that were (1) approved and already available on the market for this indication; (2) recommended by WHO, or otherwise in routine clinical use for this indication; (3) already recommended or widely used to treat a subgroup of women within the condition of interest (for example, those for use postpartum, that are contraindicated in pregnancy); (4) inactive due to negative trial outcomes (such as adverse maternal or neonatal outcomes); (5) targeted at one symptom of pre-eclampsia, rather than the underlying pathology; and (6) indicated as inferior to current treatments based on currently available evidence.

### TPP matching of candidates in development phases I, II or III

We previously developed TPPs to guide the development of novel drugs for the prevention and treatment of pre-eclampsia — the first TPPs developed for an obstetric condition [14]. These TPPs included 21 parameters with “minimum” and “preferred” criteria defined for each parameter (Additional file 1: tables S1 and S2). A hypothetical “ideal” medicine would be one that met the preferred criteria for all 21 parameters. For this study, nine of the most critical parameters from the TPPs were used as criteria to rank candidates in phases I, II or III (Table 1, Additional file 1: tables S3 and S4); these nine variables were selected based on their relative importance for wide-scale implementation, following our consultations with diverse experts (from clinical practice, research, academia, international organisations, funders, consumer representatives and representing geographical diversity) during the TPP development process. TPP matching was performed for each candidate in clinical development (defined as candidates that had been registered to be tested in human clinical trials) by two reviewers independently, and where differences arose, a third reviewer determined final matching.

**Table 1:** Critical parameters from Target Product Profiles used to rank candidates in clinical development

1. **Setting** — Has the drug been trialled for this indication in high-income country settings only, low-middle-income country settings only or both?	
2. **Efficacy** — In the available trials for this indication, has the drug demonstrated a clinically significant effect on the efficacy outcome/s?	
3. **Need for a companion diagnostic test** — In the available trials for this indication, has the drug required the routine use of a companion diagnostic test?	
4. **Need for clinical monitoring** — In the available trials for this indication, has the drug required the use of routine monitoring, or additional clinical monitoring?	
5. **Safety** — In the available trials for this indication, has the drug demonstrated any safety concerns?	
6. **Mode of administration** — In the available trials for this indication, what is the mode of administration? If no trials have been completed, what is the mode of administration for repurposed drugs?	
7. **Treatment adherence** — In the available trials for this indication, what has been the adherence to treatment?	
8. **Stability** — Is cold chain required for this product?	
9. **WHO Essential Medicines List** — Is the product currently listed on the WHO Essential Medicines List or not?	

Preclinical candidates, defined as candidates that were under investigation in non-human, laboratory settings, were assessed descriptively, including categorisation by product type, new or repurposed, and medicine subclass. A comparison of preclinical candidates to the TPPs was not performed, given the lack of clinical data available.

### Data visualisation and ranking of potential

For each variable, candidates were assigned a numerical score representing the level of matching for a given variable of the TPP (Table 1 and Additional file 1: table S3). Given the greater importance of matching to the requirements for clinical efficacy and safety, these variables were given a greater weight. These scores were also represented graphically (Figs. 3 and 4) — candidates were classified as having met preferred (dark green), met minimum (light green), partially met minimum (yellow) or did not meet minimum (red). Hence, the ranking of a candidate as high, medium or low is based on a systematic assessment of available evidence against pre-specified criteria.

## Results

Across the 153 candidates identified for prevention and/or treatment of pre-eclampsia, only one (0.7%) was approved and on the market for the treatment of pre-eclampsia/eclampsia (magnesium sulphate). An additional 13/153 (8.5%) candidates were approved for other clinical conditions and used off-label for pre-eclampsia.

**Fig. 1:**
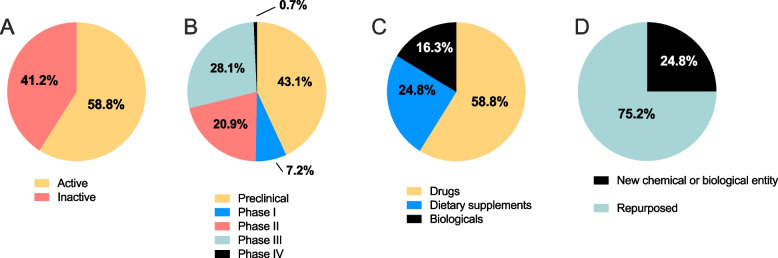
Details of the candidates in the R&D pipeline for pre-eclampsia. Summary of the 153 candidates in the R&D pipeline for the prevention and treatment of pre-eclampsia from 2000 to 2021. The proportion of candidates **A** in active development, and inactive (no publications since 2018), **B** in each phase of the development pipeline **C** classified as drugs, dietary supplements or biologicals and **D** classified as new chemical or biological entities or repurposed drugs

Of the 153 candidates, 90 (58.8%) were currently actively investigated and 63 (41.2%) were not (i.e. no published activity in the last 3 years; Fig. 1A). In total, 66 candidates were in the preclinical stage (43.1%), 11 candidates were in phase I (7.2%), 32 candidates were in phase II (20.9%), 43 candidates were in phase III (28.1%) and 1 candidate was in phase IV (0.7%; Fig. 1B). Thirty-eight candidates were classified as dietary (24.8%), 25 were biological (16.3%) and 90 were classified as drugs (58.8%; Fig. 1C). A quarter of all candidates (38 candidates, 24.8%) were new chemical/biological entities, while the remaining were repurposed (115 candidates, 75.2%; Fig. 1D).

**Fig. 2:**
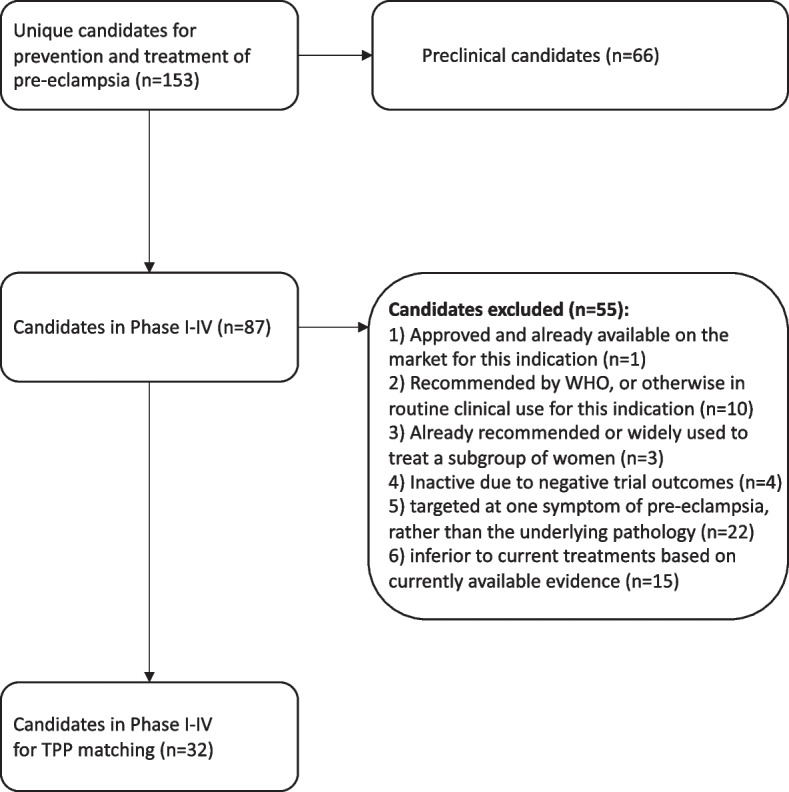
Flowchart of assessment of candidates against the eligibility criteria

Of the 87 candidates in phases I to IV, 55 were excluded from further analysis (Fig. 2, Additional file 2: table S5), leaving 32 candidates. Of these, seven were ranked as high potential, eight ranked as medium potential and 21 ranked as low potential. Some candidates were being investigated as both a treatment and a prevention agent, so the total number of candidates ranked for potential is greater than the total number of unique candidates.

### Prevention of pre-eclampsia

**Fig. 3:**
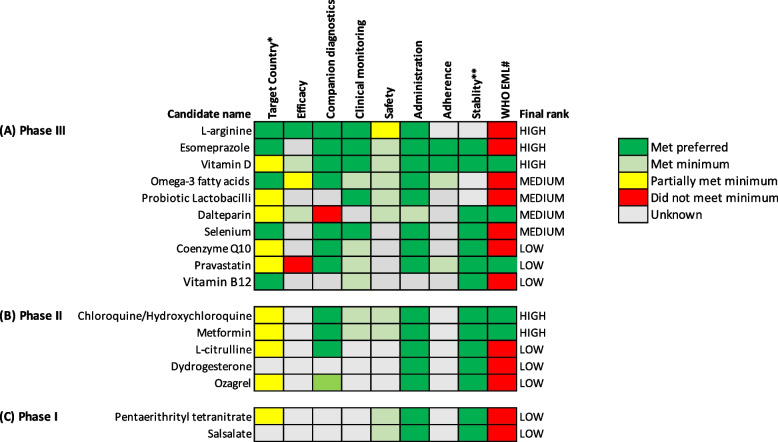
Visual representation of target product profile matching for candidates to prevent pre-eclampsia. A traffic light system to visualise each candidate for pre-eclampsia prevention at **A** phase III, **B** phase II and **C** phase I clinical development. Candidates are classified as met preferred (dark green), met minimum (light green), partially met minimum (yellow) and did not meet the minimum (red) requirements in the target product profiles. When insufficient information is available for a specific variable, they have been classified as not yet known (grey). *Target country is classified as trials being conducted in HIC and LMIC (dark green), HIC only or LMIC only (both yellow) or country not stated (grey). **Stability has been classified as does not require cold chain (green), requires cold chain (red) or unsure (grey). #WHO EML is classified as the candidate is already on the WHO EML list (green) or the candidate is not on the WHO EML list (red). The final rank has been determined by quantification of the matching to the target product profiles (see Additional file 1: tables S3 and S4 for details of quantification coding), with efficacy and safety given a greater weight than other variables. HIC high-income country, LMIC low- or middle-income country, EML essential medicines list

There were ten candidates under investigation for pre-eclampsia prevention in phase III (probiotic lactobacilli, vitamin D, omega-3 fatty acids, l-arginine, coenzyme Q10, dalteparin, esomeprazole, pravastatin, selenium and vitamin B12; Fig. 3A); five in phase II (chloroquine/hydroxychloroquine, l-citrulline, dydrogesterone, metformin, ozagrel, Fig. 3B) and two in phase I (pentaerythrityl tetranitrate and salsalate; Fig. 3C). Five candidates were ranked as high potential and four as medium potential. Results for low-priority candidates are included in supplementary data (Additional file 3).

#### Phase III candidates

Using the TPP ranking, l-arginine, esomeprazole, and vitamin D were ranked as high potential for preventing pre-eclampsia. l-Arginine, an essential amino acid, met the TPP preferred requirements for six variables, including clinical efficacy (Fig. 3A). A meta-analysis of seven l-arginine supplementation trials (884 women) conducted across high- and low-middle-income countries suggest that l-arginine may protect against pre-eclampsia and subsequent preterm birth, although the authors highlighted that further large, high-quality trials were needed [22]. Safety was only partially met, as one trial indicated a concern that women with diabetes should not consume the l-arginine supplement, which came in two nutritional bars per day [23]. However, this may be unnecessarily cautious considering there are no serious safety concerns with l-arginine in pregnancy [22, 23]. Esomeprazole, a small proton-pump inhibitor, met the TPP preferred or minimum requirements for seven variables; however, clinical efficacy remains uncertain (Fig. 3A). As of May 2021, there are 12 ongoing or completed esomeprazole trials for pre-eclampsia, three of which are investigating esomeprazole for pre-eclampsia prevention. Two trials are being conducted in Australia [24, 25] and one in Egypt [26]. Vitamin D met the preferred or minimum requirements for eight variables, including the minimum for clinical efficacy. A 2020 meta-analysis indicated that vitamin D supplementation reduced pre-eclampsia (4 trials, 499 women, RR 0.48, 95% CI 0.30–0.79; moderate-certainty evidence) [27]; however, one included trial has since been retracted [28]. In addition, a 2022 systematic review of 22 observational studies showed that women with insufficient or deficient vitamin D levels during pregnancy had higher odds of pre-eclampsia than vitamin D-replete women [29].

Three dietary supplements (omega-3 fatty acids, selenium and probiotic lactobacilli) and the low molecular weight heparin (LMWH) dalteparin were ranked as medium potential and all met the TPP requirements for most variables (Fig. 3A). Questions remain about the clinical efficacy of omega-3 fatty acids — while a 2020 meta-analysis (20 trials, 10,806 women) found that omega-3 fatty acid supplementation may reduce the risk of pre-eclampsia in women with low-risk pregnancies (RR 0.84, 95% CI 0.69–1.01) [30], a 2019 trial by Makrides et al. (which was not included in the 2020 meta-analysis) of 5517 women showed that daily fish oil capsules containing 900mg n-3 long-chain polyunsaturated fatty acids had no effect on pre-eclampsia [31]. A 2015 UK trial found that selenium supplementation in 230 pregnant women with a selenium  deficit reduced the odds of pre-eclampsia (OR 0.3, 95% CI 0.09–1.00) [32]; similar results were observed in a smaller Iranian trial of 166 pregnant women supplemented with selenium [33]. Although there is currently a lack of evidence on the clinical efficacy of probiotic lactobacilli in preventing pre-eclampsia, registered phase III trials in Sweden and the USA will further elucidate its potential and hence its ranking may change [34, 35]. Meta-analysis of LMWH trials, including three trials specifically using dalteparin, found a reduced risk of pre-eclampsia with all LMWH (797 women, RR 0.37, 95% CI 0.22–0.61) and specifically for dalteparin (190 women, RR 0.27, 95% CI 0.11–0.63), compared to no treatment [36]. The dalteparin trials required a normal thrombophilia screen (a combination of tests to identify deficiencies in a number of natural anti-coagulants) prior to inclusion. Should this companion diagnostic be required for real-world use, it may hinder the widespread implementation [37].

#### Phase II candidates

The antimalarial drug chloroquine/hydroxychloroquine and the oral antihyperglycemic agent metformin were ranked high potential, with both meeting the preferred or minimum requirements for most TPP variables. However, clinical efficacy remains unknown. A 2021 meta-analysis of seven observational studies found that hydroxychloroquine reduced the odds of pre-eclampsia in pregnant women with systemic lupus erythematosus, compared to no hydroxychloroquine (680 women, OR 0.35, 95% CI 0.21–0.59) [38]. A trial of this treatment in 50 pregnant women without systemic lupus erythematosus has also been registered [39]. A 2017 systematic review of metformin in pregnancy found five trials and four cohort studies: while there was no difference in the risk of pre-eclampsia compared to placebo, when compared to insulin, metformin was associated with lower gestational weight gain and a lower risk of pre-eclampsia [40]. A trial of metformin in 414 pregnant women with a high risk of pre-eclampsia in Qatar has been registered [41].

#### Phase I candidates

No candidates ranked high or medium potential.

### Treatment of pre-eclampsia

**Fig. 4:**
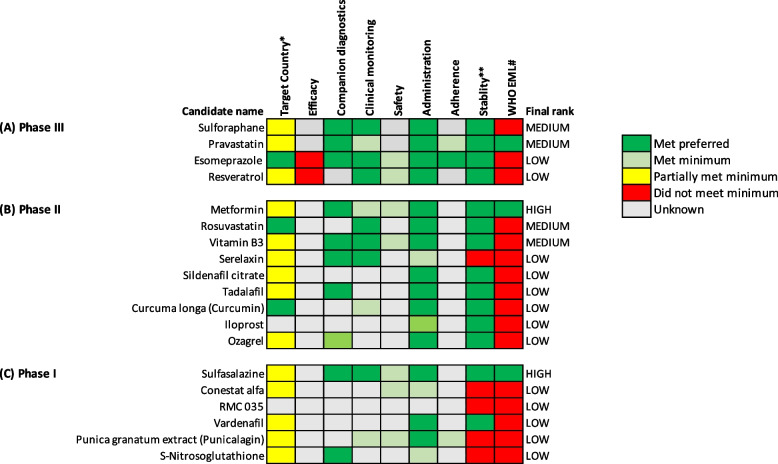
Visual representation of target product profile matching for candidates to treat pre-eclampsia. A traffic light system to visualise each candidate for pre-eclampsia treatment at **A** phase III, **B** phase II and **C** phase I clinical development. Candidates are classified as met preferred (dark green), met minimum (light green), partially met minimum (yellow) and did not meet the minimum (red) requirements in the target product profiles. When insufficient information is available for a specific variable, they have been classified as not yet known (grey). *Target country is classified as trials being conducted in HIC and LMIC (dark green), HIC only or LMIC only (both yellow) or country not stated (grey). **Stability has been classified as does not require cold chain (green), requires cold chain (red) or unsure (grey). #WHO is classified as candidate is already on the WHO EML list (green), or candidate is not on the WHO EML list (red). The final rank has been determined by quantification of the matching to the target product profiles (see Additional file 1: tables S3 and S4 for details of quantification coding), with efficacy and safety given a greater weight than other variables. HIC high-income country, LMIC low- or middle-income country, EML essential medicines list

Four candidates have been investigated for the treatment of pre-eclampsia through phase III trials (sulforaphane, esomeprazole, pravastatin and resveratrol; Fig. 4A), nine candidates in phase II (*Curcuma longa*, vitamin B3, serelaxin, sildenafil citrate, tadalafil, rosuvastatin, iloprost, ozagrel and metformin, Fig. 4B) and six in phase I (vardenafil, *Purnica granatum* extract, sulfasalazine, conestat alfa, S-nitrosoglutathione and RMC 035; Fig. 4C). Two candidates were ranked as high potential and four as medium potential. Results for low-priority candidates are included in supplementary data (Additional file 3).

#### Phase III candidates

Sulforaphane (broccoli extract) met the requirements for four TPP variables and was ranked as medium potential. Currently, the clinical efficacy, safety and adherence rates remain unknown, with a trial recruiting 180 women currently underway in Australia [42]. Pravastatin was also ranked medium as it met six of the TPP requirements, though a 2020 trial including 62 women with early-onset pre-eclampsia reported no effect of pravastatin on maternal plasma soluble fms-like tyrosine kinase-1 (sFlt-1) levels or pregnancy duration [43]. Difficulties in recruiting women may mean the study was underpowered.

#### Phase II candidates

Metformin ranked as high potential for the treatment of pre-eclampsia, having met the preferred or minimum requirements for six TPP variables*.* Clinical efficacy remains unknown, but a 2021 trial of 180 women with preterm pre-eclampsia in South Africa found that metformin significantly prolonged pregnancy by a mean of 7.6 days compared to placebo [44]. Rosuvastatin ranked as medium potential, as it met the preferred requirements for four of the variables. Clinical efficacy remains unknown and two trials are registered in Israel and Egypt [45, 46]. Vitamin B3 also ranked medium potential and met the requirements for five variables; however, clinical efficacy remains unknown, with a phase II trial in the USA registered in 2018 [47].

#### Phase I candidates

Sulfasalazine, a drug used for inflammatory bowel disease, was ranked as high potential as it met the preferred or minimum requirements for six TPP variables. The outcomes of an Australian phase I clinical trial investigating sulfasalazine as a treatment for pre-eclampsia will provide important data on clinical efficacy [48].

### Preclinical candidates

**Fig. 5:**
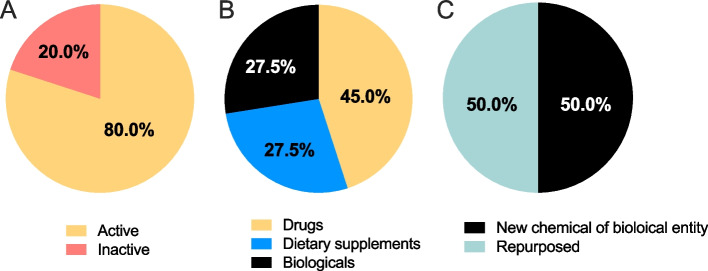
Details of the included preclinical candidates in the research and development pipeline for pre-eclampsia prevention and treatment. Summary of the preclinical candidates in the R&D pipeline for the prevention and treatment of pre-eclampsia from 2000 to 2021. The proportion of candidates **A** in active development, and inactive (no publications since 2018); **B** classified as drugs, dietary supplements or biologicals; and **C** classified as new chemical or biological entities or repurposed drugs

Of the 66 candidates in preclinical development, 26 were excluded due to potential adverse effects in humans, or the target of the candidate being unclear. From the remaining 40 candidates, 32 candidates (80%) were active and 8 (20%) were inactive (Fig. 5A). A total of 18 (45%) were drugs, 11 (27.5%) were biologics and 11 (27.5%) were dietary supplements (Fig. 5B). Repurposed medicines accounted for 50% of the preclinical candidates (20 candidates; Fig. 5C). Eleven candidates have been proposed for the prevention of pre-eclampsia (Table 2), 24 as a treatment of pre-eclampsia (Table 3) and 5 for both prevention and treatment (Tables 2 and 3).

**Table 2:** Summary of preclinical candidates for pre-eclampsia prevention

Drug subclass	Candidate	Summary	Archetype
Amino acid-peptide	l-Ergothione	Amino-acid supplement	Repurposed
Antioxidant	Hydrogen-rich saline	Alternative form of molecular hydrogen	Repurposed
Enzyme inhibitors (statins)	Simvastatin	Antilipemic agent from the statin family of drugs	Repurposed
	Lovastatin^a^	Antilipemic agent from the statin family of drugs	Repurposed
Hydrogen sulphide donors	AP39^a^	Mitochondrial-targeted small molecule	New
	MZe786	Sulphide-releasing aspirin	New
Macronutrients	Trehalose^a^	Disaccharide	Repurposed
Polyphenol	Grape seed extract	Supplement high in polyphenols	Repurposed
	Mangiferin	Non-steroidal phytopolyphenol molecular extracted from mangoes	Repurposed
	Quercetin^a^	Plant flavonoid high in polyphenols	Repurposed
	*Scutellaria baicalensis* root extract	Traditional Chinese medicinal plant extract	Repurposed
	*Uncaria rhynchophylla* extract	Traditional Chinese medicinal plant extract	Repurposed
	Vitexin	Flavonoid high in polyphenols	Repurposed
	*Vitis labrusca/vinifera* extract	Extract of the common grape vine	Repurposed
DNA, siRNA, mRNA	Ad-VEGF [viral vector delivery]^a^	Growth factor that stimulates blood vessel formation	New
Small molecule	TRV027	AT1 receptor agonist	New

^a^Candidate also under investigation for pre-eclampsia treatment

**Table 3:** Summary of preclinical candidates for pre-eclampsia treatment

Drug subclass	Candidate	Summary	Archetype
Amino acid-peptide	Cibinetide	11 amino-acid peptide	New
	Etanercept	Dimeric fusion protein that binds specifically to TNF	Repurposed
	Liraglutide	Type II diabetes and obesity medication	Repurposed
	Placental growth factor	Angiogenic growth factor	New
	SynB1-ELP-p50i [polypeptide delivery]	Drug delivery system based on the bioengineered protein ELP	New
	VEGF-B [polypeptide delivery]	Growth factor that stimulates blood vessel formation, fused with ELP	New
Antioxidant	Ferulic acid	Antioxidant found in Chinese medicinal herbs	Repurposed
	HTHQ	Potent lipophilic phenolic antioxidant	New
	MitoQ	Comprised on the antioxidant coenzyme Q and targeted lipophilic cations	Repurposed
Cell therapy	Emiplacel	Human placental stromal cell therapy	New
	Haemoglobin-vesicles [nanoparticle delivery]	Liposome encapsulated haemoglobin	New
	Regulatory T cells	Immune cells that supress autoreactive T cells	New
Enzyme inhibitors (statins)	Lovastatin^a^	Antilipemic agent from the statin family of drugs	Repurposed
Herbal	*Euterpe oleracea*	Açai fruit seed extract	Repurposed
	Toki-shakuyaku-san	Traditional Japanese Kampo medicine	Repurposed
Hydrogen sulphide donors	AP39^a^	Mitochondrial-targeted small molecule	New
	GYY4137	Sulphide-releasing aspirin	New
Macronutrients	Trehalose^a^	Disaccharide	Repurposed
Polyphenol	Quercetin^a^	Plant flavonoid high in polyphenols	Repurposed
DNA, siRNA, mRNA	AGT-targeting siRNA	Small interfering RNA therapeutic targeting angiotensinogen	New
	GPCR-AAB binding aptamers	DNA aptamer-based drugs	New
	sFlt-1-targeting siRNA	Small interfering RNA therapeutic targeting soluble fms-like tyrosine kinase-1	New
	sFlt-1-targeting siRNA [nanoparticle delivery]	P-CSA-BP-conjugated nanoparticles loaded with sFLT1 siRNA	New
	Ad-VEGF [viral vector delivery]^a^	Growth factor that stimulates blood vessel formation	New
Small molecule	Gefitinib	Potent inhibitor of sFLT1	Repurposed
	SB203580	Pyridinyl imidazole compound which inhibits MAP kinase	New
	Sofalcone	Chalcone derivative synthesised from the root of a Chinese medicinal plant (*Sophora subprostrata*)	Repurposed
Vascular agents	Carveol	Monotepene widely used in perfume, soap and shampoo	New
	Tetramethylpyrazine	Traditional Chinese medicine	Repurposed

^a^Candidate also under investigation for pre-eclampsia prevention

The most common drug subclass was amino acid/peptides (7 candidates; 17.5%) and polyphenols (7 candidates; 17.5%), followed by small interfering RNA (siRNA), messenger RNA (mRNA) or DNA-based medicines (5 candidates; 12.5%). Other medicine subclasses included antioxidants, small molecules, cell therapies, hydrogen sulphide donors, statins, herbal medicines, vascular agents and macronutrients. We identified some concerns with the preclinical efficacy evidence for half of the preclinical candidates (20 candidates). These concerns included the use of extremely high doses of the candidate in preclinical studies (8 candidates). Other concerns included results that did not show any positive effects, the use of inappropriate animal models of pre-eclampsia and the use of inappropriate controls or statistics.

## Discussion

We systematically analysed the R&D pipeline for medicines to prevent or treat pre-eclampsia over the last 20 years. Of the 153 candidates, less than 1% have made it to market for this indication and less than 9% are recommended in international guidelines or are otherwise in routine clinical use. Repurposed medicines accounted for 96% of candidates in clinical development, although 50% of candidates at the preclinical research stage were novel medicines. Through matching the candidates to pre-specified criteria derived from publicly available TPPs, five high-priority candidates for pre-eclampsia prevention (esomeprazole, l-arginine, chloroquine/hydroxychloroquine, vitamin D and metformin) and two high-priority candidates for the pre-eclampsia treatment (metformin and sulfasalazine) were identified. This is the first study in which the R&D medicines pipeline for a maternal condition has been evaluated and compared to TPPs. It offers an innovative, systematic method for identifying the best candidates for R&D investment that will meet real-world clinical needs.

The pre-eclampsia R&D pipeline is larger than that of other obstetric conditions (such as postpartum haemorrhage and foetal growth restriction); however, 63% of candidates in the clinical stages of development were excluded, predominantly due to only targeting a complication of pre-eclampsia, for example, anti-hypertensives and anti-coagulants. One of the challenges of preventing and treating pre-eclampsia is the complexity of the aetiology and presentation. Pre-eclampsia is multifactorial, involving genetic, immunological and environmental factors, and can compromise multiple organs [49]. While improving on current therapies for pre-eclampsia complications is valuable, there is arguably greater progress to be made by developing medicines that target the underlying causes of pre-eclampsia. This is particularly relevant given recent advances in understanding of the condition’s aetiology [49].

A 2020 review of proposed candidate medicines for the prevention and treatment of pre-eclampsia reported some of the candidates identified in this study, including metformin, esomeprazole, pravastatin and sulfasalazine [50]. In the current study, the process of ranking candidates against the TPP requirements provides a drug-agnostic, objective method for determining the potential of candidates. Through this process, a number of high and medium potential candidates were identified that had not been focused on in previous reviews, including l-arginine, dalteparin, chloroquine/hydroxychloroquine and probiotic lactobacilli, providing new targets for research [50, 51].

Of the 26 analysed candidates in clinical development, all but one were repurposed (i.e. drugs approved for another indication); all 12 candidates identified as high or medium potential were repurposed drugs. Some candidates (e.g. metformin) were repurposed from common comorbidities for pre-eclampsia, such as diabetes and chronic hypertension. There are advantages to repurposing drugs for maternal conditions including the availability of safety data [50]. In the ranking of potential, greater weight was given to clinical efficacy and safety, though in most cases the clinical efficacy of the candidates was not yet known — this was particularly the case for candidates at phase I or II. It is also plausible that repurposed drugs progress through clinical research phases more quickly, potentially explaining the relative lack of novel candidates in clinical development. In contrast, 50% of candidates in the preclinical phase of development were new medicines, possibly reflecting an increase in innovative research in the field. In total, 80% of preclinical candidates were active, meaning the research was published in the last 3 years. The recent advances in understanding the underlying causes of pre-eclampsia [49], coupled with rapid progress in novel molecular (RNA/DNA or protein based) medicines [52], could explain this pattern. However, it is also possible that barriers that prevent the translation of novel drug candidates into clinical trials, such as lack of funding, translational research expertise or clinical relevance of preclinical science, could be playing a role [53]. For example, we identified concerns with the clinical relevance of the published evidence for many of the preclinical candidates. Concerns most commonly related to the use of extremely high drug doses (up to 25× the safe human dose) used to demonstrate reductions in pre-eclampsia and associated symptoms in preclinical models. Whatever the reasons, the lag-time between preclinical research and translation into practice suggests that few, or no genuinely novel pre-eclampsia medicines will be available for clinical use in the near future, without significant efforts by researchers and industry to progress promising candidates to late-stage clinical trials.

Although approximately 4.6% of pregnancies are affected, pre-eclampsia meets the criteria for orphan drug designation as it qualifies as a rare disease when incidence in the total population is considered [54]. Orphan drug designation provides incentives to stimulate the development of drugs for rare diseases. A small number of the medicines identified in the database have been granted orphan drug status for pre-eclampsia or placental insufficiency, including RMC 035 and Emiplacel. Compared to other conditions, very few drugs have been granted orphan drug designation for obstetric conditions, and increased application of orphan drug designation could represent a pathway to increased investment in maternal drug development [54, 55].

Pre-eclampsia is a condition that affects pregnant women worldwide, though the prevalence and adverse consequences of pre-eclampsia are often worse for pregnant women without access to high-quality pregnancy care [4]. The TPPs for pre-eclampsia reflect this: diverse stakeholders were integral to the TPP development process, and the minimum requirements were designed to ensure plausible implementation of successful candidates in low-resource settings [14]. However, many of the candidates in the pipeline required cold-chain transport and storage, which is a known barrier to implementation in many LMICs. For example, while injectable oxytocin is effective for preventing and treating postpartum haemorrhage, its cold-chain requirements have restricted its use in settings where temperature-controlled transport and storage cannot be guaranteed [56]. As such, candidates with cold-chain requirements do not meet the TPP minimum criteria [14].

This analysis also highlighted that most pre-eclampsia candidates are not being trialled in high-, middle- and low-income countries. While some candidates had been evaluated using phase II or III trials, the rationale for investigating an individual product was often not well-articulated. Even across trials of the same intervention, doses, timing of initiation, regimens and outcomes measured differed. This may be influenced by the complex presentation of pre-eclampsia, such as early or late onset and pre-eclampsia with or without severe features. In general, it seemed arbitrary as to which candidates had or had not undergone clinical trials and where these trials had occurred, seemingly driven more by the interests of individual research groups rather than a systematic consideration of which candidates are most likely to work. If there is to be a reduction in the global burden of pre-eclampsia, an “end-to-end” approach that considers how medicine R&D can be targeted to meet the real-world needs of both high- and low-resource settings is required.

We have developed a novel, drug agnostic approach for analysing the R&D pipeline for medicines for pre-eclampsia, identifying seven high-priority candidates currently in clinical development. This approach may be useful for prioritising research for other maternal conditions, as well as other fields of drug development, particularly where TPPs already exist [16]. However, this analysis has some limitations that must be acknowledged. Firstly, ranking of candidates relied on available information identified in the AIM database, and typically repurposed candidates were ranked higher than new medicines. While the majority of candidates in clinical phases were repurposed medicines, we believe this is influenced by such medicines requiring less time to move through clinical research phases [50]. Secondly, ranking did not specifically assess the quality of the trials. The authors considered the total sum of the trial information available in the final assessment. This system of matching candidates to the TPPs was not possible for candidates in preclinical development. Thus, when examining preclinical candidates, other factors such as the quality of the laboratory findings should be considered prior to further investments in clinical trials. While our assessment is a comprehensive analysis of medicines for pre-eclampsia, food-based interventions (such as beetroot juice) or other products (such as plasma expanders) were excluded from the database. While evidence is currently limited as to the efficacy of these interventions [57, 58], products outside the scope of the AIM database may provide benefit to women with pre-eclampsia. Finally, rapid developments in the pre-eclampsia field require the database and analysis to be regularly updated as new results become available, including progress on current candidates in the pipeline, the addition of new candidates as they develop and the expansion of information collected, including additional searches based on orphan drug designation. Additionally, the TPPs are considered living documents, and any analysis of candidate in the pipeline would be reviewed following major changes to the TPP documents.

## Conclusions

We identified seven high potential and eight medium potential candidates in clinical development for pre-eclampsia prevention and treatment. A drug-agnostic, TPP matching approach was helpful in identifying the most promising candidate medicines. This novel method of comparing candidates in clinical development to the TPPs for pre-eclampsia could be applied to other maternal conditions and can help better direct research funding towards the most promising candidates for reducing morbidity and mortality due to pre-eclampsia. This methodology is also useful for designing an end-to-end development strategy for promising candidate products and could potentially reduce the time needed for downstream product introduction.

## Supplementary Information

**Additional file 1: Table S1.** Target Product Profile for medicines to prevent pre-eclampsia. **Table S2.** Target Product Profile for medicines to treat pre-eclampsia. **Table S3.** Scoring of target product profile comparison, for quantification of potential of candidates. **Table S4.** Threshold for ranking of potential at each phase of the R&D development pipeline.

**Additional file 2: Table S5.** Candidates that met the exclusion criteria for TPP matching.

**Additional file 3.** Supplementary Data - Low priority candidates.

## Data Availability

The database generated and analysed during the current study is available at https://www.policycuresresearch.org/maternal-health-pipeline/.

## Abbreviations

AIM: Accelerating Innovation for Mothers
ICTRP: International clinical trials registry platform
LMIC: Low- and middle-income countries
LWMH: Low molecular weight heparin
R&D: Research and development
sFlt-1: Soluble fms-like tyrosine kinase-1
TPP: Target product profile
WHO: World Health Organization
