# Supplementary material for: Systematic evaluation of the pre-eclampsia drugs, dietary supplements and biologicals pipeline using target product profiles
Source: BMC Med. 2022 Nov 4;20:393. doi: 10.1186/s12916-022-02582-z (PMC9635102; doi:10.1186/s12916-022-02582-z)
Supplement: Supplementary file 1 — Additional file 1: Table S1. Target Product Profile for medicines to prevent pre-eclampsia. Table S2. Target Product Profile for medicines to treat pre-eclampsia. Table S3. Scoring of target product profile comparison, for quantification of potential of candidates. Table S4. Threshold for ranking of potential at each phase of the R&D development pipeline. [file 12916_2022_2582_MOESM1_ESM.docx]

**Additional file 1: Supplementary Tables S1 – S4**

Table S1. Target Product Profile for medicines to prevent pre-eclampsia

|  | **Minimum**  *The minimal target should be considered as a potential go/no go decision point.* | **Preferred**  *The preferred (or optimistic) target should reflect what is needed to achieve broader, deeper, quicker global health impact.* | **Annotations**  *For all parameters, include here the* ***source data used and rationale*** *for why this feature is important.* |
| --- | --- | --- | --- |
| **Indication** | Prophylactic treatment of pregnant women at increased risk of developing pre-eclampsia. | Same as minimum | The medicine is intended to prevent pre-eclampsia in pregnant women at increased risk, to improved maternal and fetal/neonatal mortality and morbidity outcomes. |
| **Target population** | Pregnant women with identified risk factors for pre-eclampsia. | Same as minimum | There is currently a lack of consensus on the criteria for identifying women at risk of pre-eclampsia. WHO recommendations(4) define the risk factors as:  *moderate risk*: any two of the following risk factors: primiparity, family history of pre-eclampsia, age greater than 40 years, or multiple pregnancy.  *high risk:* one or more of the following risk factors: diabetes, obesity, chronic or gestational hypertension, renal disease, autoimmune disease, positive uterine artery Doppler, previous history of pre-eclampsia, or previous fetal/neonatal death associated with pre-eclampsia.  The recommendations note that this not an exhaustive list of risk factors and can be adapted based on the local epidemiology of pre-eclampsia. |
| **Special populations** | Safe and effective in women with common co-morbidities (e.g., chronic hypertension, type I or II diabetes, obesity, chronic kidney disease or autoimmune disease) and in pregnant adolescents (<18 years old). | Safe and effective in all pregnant women. | The population of women at increased risk of pre-eclampsia are also more likely to have other co-morbidities, including chronic hypertension, type I or II diabetes, chronic kidney disease or autoimmune disease. |
| **Population unlikely to be treated** | Women with a medical contraindication to the preventive agent.  Women currently diagnosed with pre-eclampsia or eclampsia. | Same as minimum. |  |
| **Target countries** | All high-, middle- and low-income countries | Same as minimum | The incidence of pre-eclampsia and eclampsia is estimated at 4.6% and 1.4% of pregnant women, respectively.(1)  Approximately 16% of pregnant women in the UK are at an increased (moderate – high) risk of pre-eclampsia.(53) |
| **Efficacy** | Clinically significant reduction in pre-eclampsia incidence, or delayed onset of pre-eclampsia in women at increased risk. | Clinically significant reduction in pre-eclampsia incidence, or delayed onset of pre-eclampsia in women at increased risk.  Clinically significant reduction in serious adverse maternal or fetal/neonatal outcomes associated with pre-eclampsia | WHO recommends that women at moderate or high risk of pre-eclampsia should be treated with daily low-dose aspirin as a preventive therapy. Based on evidence from 60 studies, aspirin probably reduces the risk of pre-eclampsia by 18% (RR 0.82, 95% CI 0.77 – 0.88).(54) |
| **Is companion diagnostic needed for use?** | No. Identifying women at risk of pre-eclampsia requires a thorough history and clinical examination.  Some conditions that increase risk of pre-eclampsia require use of special tests.  *moderate risk*: any two of the following risk factors: primiparity, family history of pre-eclampsia, age greater than 40 years, or multiple pregnancy.  *high risk:* one or more of the following risk factors: diabetes, obesity, chronic or gestational hypertension, renal disease, autoimmune disease, positive uterine artery Doppler, previous history of pre-eclampsia, or previous fetal/neonatal death associated with pre-eclampsia. | Same as minimum. | A number of risk factors have been identified as increasing risk of pre-eclampsia many of which are identified based on history and examination, though some (such as gestational diabetes or positive uterine artery Doppler, angiogenic factors) require special tests. |
| **Need for clinical monitoring?** | Regular clinical assessments as part of standard care for women at risk of pre-eclampsia, including monitoring for fetal health and well-being.  Minimal additional monitoring required for expected drug side-effects. | Regular clinical assessments as part of standard care for women at risk of pre-eclampsia, including monitoring for fetal health and well-being.  No additional monitoring required for expected drug side-effects. | Women at risk of pre-eclampsia should be regularly assessed in antenatal care settings to identify signs or symptoms of pre-eclampsia. |
| **Clinical endpoint for licensure** | Reduced incidence of pre-eclampsia amongst pregnant women at increased risk | Reduced incidence of pre-eclampsia  Reduced incidence of adverse maternal and fetal/neonatal outcomes associated with pre-eclampsia. | Clinical endpoints have been selected based on primary outcomes in Cochrane reviews of current preventative treatments for pre-eclampsia, and priority outcomes used in WHO guidelines on preventing pre-eclampsia.(4, 54) |
| **Safety** | No significant clinical adverse effects for mother and baby.  Not contraindicated in pregnant and lactating women.  Absence of embryo-fetal toxicity or teratogenicity. | No clinical adverse effects for mother and baby.  No drug-related serious adverse events for mother or baby.  Not contraindicated in pregnant and lactating women.  Absence of embryo-fetal toxicity or teratogenicity.  Evidence shows no long-term adverse effects for mothers or babies. |  |
| **Drug interactions** | No significant drug-drug interactions with common antenatal treatments (medicines or supplements), medicines used in women with pre-eclampsia (such as anti-hypertensives, antibiotics, magnesium sulfate, tocolytics or corticosteroids), or drugs used for common co-morbidities (including chronic hypertension, type I or II diabetes, obesity, chronic kidney disease or autoimmune disease). | No drug-drug interactions with common antenatal treatments (medicines or supplements), medicines used in women with pre-eclampsia (such as anti-hypertensives, antibiotics, magnesium sulfate, tocolytics or corticosteroids) or drugs used for common co-morbidities (including chronic hypertension, type I or II diabetes, obesity, chronic kidney disease or autoimmune disease). | Preventive agent will be used alongside usual antenatal care for women at increased risk of pre-eclampsia. Hence, the treatment must have minimal to no adverse interactions with drugs commonly used in pregnant women and women with pre-eclampsia. |
| **Formulation dosage & administration** | Non-invasive (including oral, inhaled, vaginal or transdermal) or injectable (IM or SC).  Treatment can be commenced early in pregnancy (e.g.: prior to 20 weeks’ gestation) and can be continued throughout pregnancy, and into the postpartum period, as required.  Regimen (dose and duration) dependent on clinical response to preventive agent. | Oral  Treatment can be commenced early in pregnancy (e.g.: prior to 20 weeks’ gestation) and can be continued throughout pregnancy and into the postpartum period, as required.  Regimen (dose and duration) dependent on clinical response to preventive agent. | Current therapies are orally self-administered.  Current novel technologies and therapies in development for pre-eclampsia prevention include non-systemic, targeted, injectables.(48)  Oral administration is preferred, and will promote acceptability, self-administration and adherence in line with current therapies.  Oral administration would likely be more feasible and acceptable for low-resource settings. |
| **Treatment adherence** | Frequency of discontinuation during therapy <30% | Frequency of discontinuation during therapy <20% | Large multi-center trials of aspirin and calcium supplements during pregnancy have reported that high intake adherence rates (>80-90%) are required for improved health outcomes. Discontinuation rates are reported as <20%.(55, 56) Treatment adherence rates do not take into consideration access to healthcare services or supplies. |
| **Stability / Shelf life** | Stable at 30°C.  Easy to transport and store.  2-year shelf life in climatic zone IVb (simulated with 30°C and 75% relative humidity).  *Biologicals:* cold-chain (2-8°C) acceptable. | Stable at 30°C.  Easy to transport and store.  3 to 5-year shelf life in climatic zone IVb (simulated with 30°C and 75% relative humidity, plus 6 months at 40°C and 75% relative humidity).  *Biologicals:* cold-chain (2-8°C) acceptable. | Given the greater burden of pre-eclampsia in LMICs, ease of transport and storage, as well as stability in hotter or humid conditions is a priority.(2) |
| **Product presentation** | Easy to open and administer.  Packaging must aim to protect and preserve the quality of the product and prevent damage to the drugs during transport and storage.  *Injectable*: packaging must maintain sterility. | Compact, lightweight, easy to open and administer, sustainable packaging.  Packaging must aim to protect and preserve the quality of the product and prevent damage to the drugs during transport and storage.  *Injectable*: packaging must maintain sterility.  Environmental impact of the packaging should be minimized. | An easy to open and administer presentation will aid in the implementation of the preventive agent, as there will be minimal additional training requirements for healthcare workers or women to self-administer.  Packaging and design must comply with regulatory guidance from a stringent regulatory authority or WHO standards. |
| **Target product registration pathway(s)** | Approval by at least 1 stringent regulatory authority (e.g., US Food and Drug Administration, European Medicines Agency)  Approval from relevant national regulatory authorities will also be required | Approval by at least 1 stringent regulatory authority (e.g., US Food and Drug Administration, European Medicines Agency)  Approval from relevant national regulatory authorities will also be required  WHO pre-qualification approval obtained | Use of a preventive agent in a given LMIC will require approval from their national regulatory authority.  Product registration pathways are likely to differ for repurposed compared to novel drug treatments.  Engaging with regulatory authorities early to discuss potential regulatory pathways and streamline the approval process is advised. |
| **WHO prequalification** | WHO listed authority application pathways within 12 months of Essential Medicines List (EML) inclusion. | WHO prequalification submission to be made within 12 months of Essential Medicines List (EML) inclusion. | WHO PQ eligibility follows guideline and EML inclusion. |
| **Primary target delivery channel** | *All:* Antenatal, childbirth and postpartum care settings (including community healthcare settings) where women at risk of pre-eclampsia receive care.  *Non-invasive:* Staff available to provide and advise women on using medicine correctly  *Injectable:* Staff, supplies and equipment available and authorised to administer medicine | *All:* Antenatal, childbirth and postpartum care settings (including community healthcare setting) where women at risk of pre-eclampsia receive care.  *Oral:* Staff available to provide and advise women on using medicine correctly | It is anticipated that the preventive agent will be used in antenatal care settings, particularly those where higher-risk women receive care. |
| **Target affordable pricing / procurement** | Preventive agent is affordable in the public sector in LMICs | Preventive agent is affordable in the public sector in LMICs    Unit cost of treatment is similar to other preventative therapies for women at increased risk of pre-eclampsia | Given the burden of pre-eclampsia in LMICs, affordability of any novel treatments is a high priority.  Current preventive agents for women with pre-eclampsia (aspirin; calcium supplements) are generally widely available and affordable. |
| **Expected financing source** | Procurement in LMICs financed by national governments, international agencies (including UN organizations), and /or international donors, or private sector | Procurement financed by national governments or private sector | Procurement of medicines for use in pregnancy in LMICs varies between countries, but it may include governments as well as support from international organizations, agencies or funders.  For a new treatment, initial support from international organizations maybe required.    Procurement of effective treatments would ideally be prioritized by national governments. |
| **Volume estimates** | Volumes compatible with incidence of pre-eclampsia | Same as minimum | The estimated global incidence of pre-eclampsia is approximately 5%, equating to ~7 million women worldwide each year (though this may be an underestimate).(1)  Limited data exists on the proportion of women who are at increased risk of pre-eclampsia, however, observational data from the UK report 16.1% of pregnant women have identified risk factors for pre-eclampsia.(53)  There are currently no reliable global estimates on the coverage of current preventative therapies for pre-eclampsia, though they are widely used. |

Table S2. Target Product Profile for medicines to treat pre-eclampsia

|  | **Minimum**  *The minimal target should be considered as a potential go/no go decision point.* | **Preferred**  *The preferred (or optimistic) target should reflect what is needed to achieve broader, deeper, quicker global health impact.* | **Annotations**  *For all parameters, include here the* ***source data used and rationale*** *for why this feature is important.* |
| --- | --- | --- | --- |
| **Indication** | Treatment of women with suspected or confirmed pre-eclampsia, regardless of severity. | Same as minimum | A therapeutic target is intended to treat pre-eclampsia in pregnant or postpartum women, and improve maternal, fetal and/or neonatal mortality and morbidity outcomes.  Typically, more severe disease is associated with worse outcomes for mother and baby. Treatment initiated early in disease progression (e.g., in women with mild disease) could potentially have greater benefits. |
| **Target population** | Pregnant and postpartum women with suspected or confirmed pre-eclampsia, regardless of severity | Same as minimum | ICD-11 characterises pre-eclampsia as the new onset of hypertension (systolic blood pressure ≥140 mmHg and/or diastolic blood pressure ≥90mmHg) and proteinuria OR significant end-organ dysfunction after 20 weeks of gestation.  As the resources for diagnosing pre-eclampsia may not always be available (particularly in low-resource settings), an agent that is effective in women with suspected pre-eclampsia would be more practical to implement across LMICs. |
| **Special populations** | Safe and effective in women who are candidates for immediate delivery (for example, those with severe symptoms of pre-eclampsia or eclampsia, fetus showing signs of distress or severe IUGR), treated to prevent postpartum pre-eclampsia.  Safe and effective in women with common co-morbidities (e.g., chronic hypertension, type I or II diabetes, obesity, chronic kidney disease or autoimmune disease), and in pregnant adolescents (<18 years old). | Safe and effective in women who are candidates for immediate delivery (for example, those with severe symptoms of pre-eclampsia or eclampsia, fetus showing signs of distress or severe IUGR), treated to prevent post-partum pre-eclampsia.  Safe and effective in all pregnant or postpartum women with any form of pre-eclampsia, including those diagnosed with HELLP syndrome or pre-eclampsia superimposed upon chronic hypertension.  Safe and effective in women with common co-morbidities (e.g., chronic hypertension, type I or II diabetes, obesity, chronic kidney disease or autoimmune disease), and in pregnant adolescents (<18 years old). | The target product profile for novel pre-eclampsia treatment is already targeting to a “special population” – pregnant and postpartum women. The optimal requirements would deliver a safe and effective treatment for pre-eclampsia in all women, including those with HELLP syndrome or pre-eclampsia superimposed upon chronic hypertension or other medical conditions allowing for delivery of the intervention in settings where the accurate differentiation between pre-eclampsia subtype was not efficient. |
| **Population unlikely to be treated** | Women with a medical contraindication to the therapeutic agent. | Same as minimum |  |
| **Target countries** | All high-, middle- and low-income countries | Same as minimum | The incidence of pre-eclampsia and eclampsia is estimated at 4.6% and 1.4% of pregnant women, respectively.(1) |
| **Efficacy** | Clinically significant difference in extending pregnancy duration to increase fetal maturity in women with preterm pre-eclampsia.  OR  Clinically significant reduction in serious adverse maternal antenatal or postpartum outcomes associated with pre-eclampsia disease progression (such as mortality, severe-pre-eclampsia, eclampsia, stroke, etc.);  OR  Clinically significant reduction in adverse fetal/neonatal outcomes associated with pre-eclampsia, (such as stillbirth, IUGR, preterm birth, neonatal mortality, admission to the NICU or other pre-eclampsia-related neonatal complications). | Clinically significant difference in extending pregnancy duration to increase fetal maturity in women with preterm pre-eclampsia.  AND  Clinically significant reduction in serious adverse maternal antenatal or postpartum outcomes associated with pre-eclampsia disease progression (such as mortality, severe-pre-eclampsia, eclampsia, stroke, etc.);  AND  Clinically significant reduction in adverse fetal/neonatal outcomes associated with pre-eclampsia, (such as stillbirth, IUGR, preterm birth, neonatal mortality, admission to the NICU or other pre-eclampsia-related neonatal complications). | Efficacy outcomes have been selected based on priority outcomes in the WHO guidelines for treating women with pre-eclampsia, and the core outcome set for pre-eclampsia.(4, 57) |
| **Is companion diagnostic needed for use?** | The International Classification of Diseases (ICD-11) describes pre-eclampsia as characterised by systolic blood pressure greater than 140mmHg or diastolic blood pressure greater than 90mmHg on two occasions, 4 hours or more apart in the presence of either proteinuria or other new onset maternal organ dysfunction, neurological conditions or fetal growth restriction.^38^  Proteinuria testing or special tests for organ dysfunction may be required for diagnosis. | Same as minimum | Special tests may be required for pre-eclampsia to be diagnosed.  Proteinuria is diagnosed through urinalysis for protein in urine. Additional diagnostic tests include laboratory evaluation of platelet count, serum creatine and liver chemistries.(58)  Other special tests (such as placental angiogenic factor-based testing) may be used for pre-eclampsia diagnosis in some settings. However, these are not widely available in LMICs and should not be regarded as a minimum requirement. |
| **Need for clinical monitoring** | Continued monitoring of maternal, fetal and neonatal health and well-being. For women treated in the postpartum period only, additional monitoring of newborns (beyond routine practice) is not required.  Minimal additional monitoring required for expected drug side-effects. | Continued monitoring of maternal, fetal and neonatal health and well-being. For women treated in the postpartum period only, additional monitoring of newborn (beyond routine practice) would not be required.  No additional monitoring required for expected drug side-effects. | Expectant management of women with pre-eclampsia includes regular monitoring of maternal blood pressure, as well as platelet count, serum creatinine and liver chemistries. Fetal growth and well-being also needs to be regularly assessed.(4) |
| **Clinical Endpoint for Licensure** | Clinically important difference in extending pregnancy duration to increase fetal maturity.  Reduced maternal clinical endpoints: death or major maternal morbidity (eclampsia, recurrent seizures, stroke, Pulmonary oedema, emergency caesarean, placental abruption etc.)  Reduced fetal/neonatal endpoints: stillbirth, neonatal death or major neonatal morbidity (IUGR, preterm birth, low birthweight, NICU admission, respiratory distress syndrome Intraventricular haemorrhage, etc.) | Same as minimum | Clinical endpoints have been selected based on priority outcomes in the WHO guidelines for treating women with pre-eclampsia, and the pre-eclampsia core outcome set.(4, 57) |
| **Safety** | Clinical safety (adverse or serious adverse effects for mother and baby) comparable to current therapies.  Not contraindicated in pregnant and lactating women.  Absence of fetal toxicity. | Fewer adverse effects than current therapies.  No drug-related serious adverse events for mother or baby.  Not contraindicated in pregnant and lactating women.  Absence of fetal toxicity.  Evidence shows no long-term adverse effects for mothers or babies. | Current treatments for specific manifestations of pre-eclampsia include antihypertensive drugs (e.g., methyldopa or labetalol) and magnesium sulfate. Drug options recommended by WHO for managing hypertensive disorders or pregnancy largely have acceptable safety profiles, though some lack evidence for fetal safety outcomes.(59, 60)  Side effects of different anti-hypertensive drugs in pregnancy vary. For example, beta-blockers can cause oedema, postural hypotension, bradycardia, cold extremities, rashes, sweating, tachycardia, nausea, dyspepsia, vomiting and difficulty in micturition.(61) Side effects of magnesium sulfate include flushing, nausea and/or vomiting, slurred speech, muscle weakness, hypotension, dizziness, drowsiness or confusion, and headache.(62) |
| **Drug interactions** | No significant drug-drug interactions with common antenatal treatments (medicines or supplements) or drugs used in women with pre-eclampsia (such as anti-hypertensives, antibiotics, magnesium sulfate, tocolytics or corticosteroids), or drugs used for common co-morbidities (including chronic hypertension, type I or II diabetes, obesity, chronic kidney disease or autoimmune disease) | No drug-drug interactions with common antenatal treatments (medicines or supplements) or with drugs used in women with pre-eclampsia (such as anti-hypertensives, antibiotics, magnesium sulfate, tocolytics or corticosteroids), or drugs used for common co-morbidities (including chronic hypertension, type I or II diabetes, obesity, chronic kidney disease or autoimmune disease). | The treatment must have minimal to no adverse interactions with drugs commonly used in pregnant or postpartum women with pre-eclampsia |
| **Formulation Dosage & Administration** | Non-invasive (including oral, inhaled, vaginal or transdermal) or parenteral (including intramuscular, intravenous or infusion)  Regimen (dose and duration) dependent on clinical response to treatment and severity of pre-eclampsia. | Oral  Regimen (dose and duration) dependent on clinical response to treatment and severity of pre-eclampsia. | Current interventions for women with pre-eclampsia are delivered either orally or parenterally, as are experimental treatments being investigated for pre-eclampsia treatment in ongoing clinical trials.(4, 48)  Oral administration is preferred, as it would likely be more feasible and acceptable in low-resource settings, particularly in settings with limited capacity to administer and monitor women receiving infusions. |
| **Treatment adherence** | Frequency of discontinuation during therapy <20% | Frequency of discontinuation during therapy <10% | Large multi-center trials of magnesium sulfate and oral antihypertensives during pregnancy have reported discontinuation rates less than 3%.(63, 64) Treatment adherence rates do not take into consideration access to healthcare services or supplies. |
| **Stability / Shelf Life** | Stable at 30°C  Easy to transport and store.  2-year shelf life in climatic zone IVb (simulated with 30°C and 75% relative humidity).  *Biologicals:* cold-chain (2-8°C) acceptable. | Stable at 30°C  Easy to transport and store.  3 to 5-year shelf life in climatic zone IVb (simulated with 30°C and 75% relative humidity plus 6-month stability at 40°C and 75% relative humidity).  *Biologicals:* cold-chain (2-8°C) acceptable. | Given the greater burden of pre-eclampsia in LMICs, ease of transport and storage, as well as stability in hotter or humid conditions is a priority.(2) |
| **Product Presentation** | Easy to open and administer.  Packaging must aim to protect and preserve the quality of the product and prevent damage to the drugs during transport and storage.  *Injectable*: packaging must maintain sterility. | Compact, lightweight, easy to open and administer, sustainable packaging.  Packaging must aim to protect and preserve the quality of the product and prevent damage to the drugs during transport and storage.  *Injectable*: packaging must maintain sterility.  Environmental impact of the packaging should be minimized. | An easy to open and administer presentation will aid in the implementation of the novel treatment, as there will be minimal additional training requirements for healthcare workers.  Packaging and design must comply with regulatory guidance from a stringent regulatory authority or WHO standards. |
| **Target Product Registration Pathway(s)** | Approval by at least 1 stringent regulatory authority (e.g., US Food and Drug Administration, European Medicines Agency)  Approval from relevant national regulatory authorities will also be required | Approval by at least 1 stringent regulatory authority (e.g., US Food and Drug Administration, European Medicines Agency)  Approval from relevant national regulatory authorities will also be required  WHO pre-qualification approval obtained | Use of a treatment in a given LMIC will require approval from their national regulatory authority.  Product registration pathways are likely to differ for repurposed compared to novel drug treatments.  Engaging with regulatory authorities early to discuss potential regulatory pathways and streamline the approval process is advised. |
| **WHO Prequalification** | WHO listed authority application pathways within 12 months of Essential Medicines List (EML) inclusion. | WHO prequalification submission to be made within 12 months of Essential Medicines List (EML) inclusion. | WHO PQ eligibility follows guideline and EML inclusion. |
| **Primary Target Delivery Channel** | *All:* Antenatal, childbirth and postpartum care settings where women with pre-eclampsia are managed and monitored.  *Non-invasive:* Staff available to administer oral treatment  *Parenteral (including infusion):* Staff, supplies and equipment available and authorised to administer parenteral treatment | *All:* Antenatal, childbirth and postpartum care settings where women with pre-eclampsia are managed and monitored.  *Oral:* Staff available to administer oral treatment | At a minimum, the treatment (oral or parenteral) would be delivered in settings with the capacity to deliver that treatment and monitor maternal and fetal well-being. |
| **Target Affordable Pricing / Procurement** | Treatment is affordable in the public sector in LMICs | Treatment affordable in the public sector in LMICs    Unit cost of treatment is similar to other treatments for women with pre-eclampsia | Given the burden of pre-eclampsia in LMICs, affordability of any novel treatments is a high priority and an integral part of access planning.  Current treatments for women with pre-eclampsia (antihypertensive drugs; magnesium sulfate) are generally widely available and affordable. |
| **Expected Financing Source** | Procurement in LMICs financed by national governments, international agencies (including UN organizations), and /or international donors, or private sector | Procurement financed by national governments or private sector | Procurement of medicines for use in pregnancy in LMICs varies between countries, but it may include governments as well as support from international organizations, agencies or funders.  For a new treatment, initial support from international organizations or donors may be required.    Procurement of effective treatments would ideally be prioritized by national governments. |
| **Volume estimates** | Volumes compatible with incidence of pre-eclampsia | Same as minimum | The estimated global incidence of pre-eclampsia is approximately 5%, equating to ~7 million women worldwide each year (though this may be an underestimate).(1)  There are currently no reliable global estimates on the coverage of current pre-eclampsia treatments in pregnancy, though they are widely used. |

Table S3: Scoring of target product profile comparison, for quantification of potential of candidates.

| **Variable** | **Answer options** | **Coding value** |
| --- | --- | --- |
| Target country | Not stated | 0 |
|  | HIC only | 1 |
|  | LMIC only | 1 |
|  | HIC and LMIC | 2 |
| Efficacy | Not yet known | 0 |
|  | Not met minimum | AUTOMATIC LOW |
|  | Partially met minimum | 1 |
|  | Met minimum | 3 |
|  | Met preferred | 5 |
| Safety | Not yet known | 0 |
|  | Not met minimum | AUTOMATIC LOW |
|  | Partially met minimum | 1 |
|  | Met minimum | 2 |
|  | Met preferred | 3 |
| Companion Diagnostic, | Not yet known | 0 |
| Monitoring, | Not met minimum | -2 |
| Adherence | Partially met minimum | 1 |
| and Administration | Met minimum | 2 |
|  | Met preferred | 3 |
| Stability (is cold chain storage required?) | Yes | -2 |
|  | No | 2 |
|  | Unsure | 0 |
| WHO EML (is candidate currently on the EML list?) | Yes | 1 |
|  | No | -1 |
|  | Unsure | 0 |

Table S4: Threshold for ranking of potential at each phase of the R&D development pipeline

| **Final ranking** | **Phase III** | **Phase II** | **Phase I** |
| --- | --- | --- | --- |
| *High potential* | >17 | >13 | >13 |
| *Medium potential* | 9-16 | 9-13 | 6-13 |
| *Low potential* | <9 | <9 | <6 |
