# Supplementary material for: Systematic evaluation of the pre-eclampsia drugs, dietary supplements and biologicals pipeline using target product profiles
Source: BMC Med. 2022 Nov 4;20:393. doi: 10.1186/s12916-022-02582-z (PMC9635102; doi:10.1186/s12916-022-02582-z)
Supplement: Supplementary file 2 — Additional file 2: Table S5. Candidates that met the exclusion criteria for TPP matching. [file 12916_2022_2582_MOESM2_ESM.docx]

**Additional file 2:**

**Supplementary table S5. Candidates that met the exclusion criteria for TPP matching**

| **Exclusion criteria** | **Candidate** | **Drug subclass** |
| --- | --- | --- |
| Approved and already available on the market for this indication | Magnesium sulfate | Anticonvulsant |
| Recommended by WHO, or otherwise in routine clinical use for this indication | Aspirin | Anti-platelet |
|  | Atenolol | Antihypertensive |
|  | Captopril | Antihypertensive |
|  | Clonidine | Antihypertensive |
|  | Labelatol | Antihypertensive |
|  | Methyldopa | Antihypertensive |
|  | Metoprolol | Antihypertensive |
|  | Calcium | Micronutrient |
|  | Nifedipine | Antihypertensive |
|  | Sodium nitroprusside | Antihypertensive |
| Already recommended or widely used for a subgroup of women | Enalapril (postpartum only) | Antihypertensive |
|  | Amlodipine (postpartum only) | Vascular agent |
|  | Furosemide (postpartum only) | Antihypertensive |
| Inactive due to negative trial outcomes | Drotrecogin alpha | Amino-acid/peptide |
|  | Estrodial | Amino-acid/peptide |
|  | Losartan | Antihypertensive |
|  | Lycopene | Micronutrient |
| Targeted at one symptom of pre-eclampsia, rather than the underlying pathology | 17-alpha-hydroxyprogesterone caproate | Amino-acid/peptide |
|  | Antithrombin gamma | Anticoagulant |
|  | Antithrombin III | Anticoagulant |
|  | Benazepril | Antihypertensive |
|  | Diltiazem | Antihypertensive |
|  | Eculizumab | Amino-acid peptide |
|  | Glyceryl-trinitrate | Vascular agent |
|  | Hydralazine | Antihypertensive |
|  | Hydrochlorothiazide | Antihypertensive |
|  | Isosorbide dinitrate | Vascular agent |
|  | Ketanserin | Antihypertensive |
|  | Levetiracetam | Anti-convulsant |
|  | Methylprednisolone | Steroid hormone |
|  | Magnesium | Micronutrient |
|  | Nicardipine | Antihypertensive |
|  | Nimodipine | Antihypertensive |
|  | Camelia sinensis (Epigallocatechin gallate) | Polyphenol |
|  | Silybum marianum (Silibinin) | Polyphenol |
|  | Oral progesterone | Amino-acid peptide |
|  | Ropivacaine | Anesthetic |
|  | Ulinastatin | Enzyme inhibitor |
|  | Urapidil | Antihypertensive |
| Inferior to current treatments based on currently available evidence | AMAG 423 | Amino-acid/peptide |
|  | Antithrombin alpha | Anticoagulant |
|  | Celecoxib | Anti-inflammatory |
|  | Enoxaparin | Anticoagulant |
|  | Garlic extract | Herbal |
|  | Isosorbide mononitrate | Vascular agent |
|  | Melatonin | Amino-acid/peptide |
|  | Nadroparin | Anticoagulant |
|  | Phenytoin | Anti-convulsant |
|  | Salvia miltiorrhiza (Salvianolic acid A) | Polyphenol |
|  | Vitamin A | Micronutrient |
|  | Vitamin B9 | Micronutrient |
|  | Vitamin C | Micronutrient |
|  | Vitamin E | Micronutrient |
|  | Zinc | Micronutrient |
