# Supplementary material for: Systematic evaluation of the pre-eclampsia drugs, dietary supplements and biologicals pipeline using target product profiles
Source: BMC Med. 2022 Nov 4;20:393. doi: 10.1186/s12916-022-02582-z (PMC9635102; doi:10.1186/s12916-022-02582-z)
Supplement: Supplementary file 3 — Additional file 3. Supplementary Data - Low priority candidates. [file 12916_2022_2582_MOESM3_ESM.docx]

**Additional file 3**

**Supplementary Data: Low priority candidates**

**Prevention of pre-eclampsia**

*Phase III*

Pravastatin, Coenzyme Q10 (ubidecarenone), resveratrol and Vitamin B12 all ranked low, despite meeting the TPP preferred requirements for most of the variables. A 2021 clinical trial of pravastatin (1120 women) did not show any evidence of efficacy for prevention of term pre-eclampsia, however pravastatin was started at 35-37 weeks’ gestation, which may be too late for prevention.(65) The clinical efficacy of Coenzyme Q10 also remains unknown, as there has been no further clinical development following a single clinical trial of 235 women, which found supplementation with Coenzyme Q10 reduced the risk of pre-eclampsia compared to placebo (RR 0.56, 95% CI 0.33 – 0.96).(66) In addition, some safety concerns have been raised about the use of MitoQ in early pregnancy, a product that contains Coenzyme Q10. MitoQ exacerbates the pre-eclampsia phenotype in rodent models when given in early gestation as it disrupts placental formation due to its antioxidant activity.(67) A 2021 systematic review of 19 observational studies examining maternal serum Vitamin B12 levels in women with and without pre-eclampsia suggests an association between Vitamin B12 deficiency and pre-eclampsia, although results were highly heterogenous.(68) The results of trials registered in Norway (NCT03071666) and China (ChiCTR1900021531) will provide further evidence of the clinical efficacy of Vitamin B12 supplementation.

*Phase II*

Dydrogesterone (a progesterone supplement), Ozagrel and L-citrulline were ranked low potential following comparison to the TPP, as it was unclear whether or not they met the requirements for most variables. There have been no clinical trials specifically reporting dydrogesterone as a preventive agent for pre-eclampsia, and a 2006 Cochrane review examining the risk of pre-eclampsia following progesterone supplementation found insufficient evidence that progesterone effected the risk of pre-eclampsia (three trials; 1277 women; RR 1.25, 95% CI 0.78 – 2.31).(69) However, large observational studies from women taking dydrogesterone supplements while using assisted reproductive technology show a significant decrease in the incidence of pre-eclampsia.(70-72) The CITRUPE trial, investigating L-citrulline supplementation as a preventive agent for severe pre-eclampsia was completed in 2021, but has not yet reported findings.(73)

*Phase I*

Both the non-steroidal anti-inflammatory salsalate and the nitric oxide donor pentaerythrityl tetranitrate ranked low following comparison to the TPP. While they both met the preferred requirements for administration and stability, and the minimum requirements for safety, it is unclear whether they meet requirements for other variables.

**Treatment of pre-eclampsia**

*Phase III*

Esomeprazole and resveratrol ranked low potential, as neither met the minimum requirements for clinical efficacy. A 2018 trial in 120 women with preterm pre-eclampsia (pre-eclampsia developing before 37 weeks’ gestation) suggested no effect of esomeprazole on maternal plasma sFlt-1 levels or prolongation of pregnancy.(74) However, the dose used may have been too low to elicit a response, and trial authors cannot rule out that higher doses of esomeprazole may be effective or the trial being underpowered.(74) There are nine ongoing or completed trials investigating esomeprazole in women with diagnosed pre-eclampsia, in China (ChiCTR1900026972), South Africa (PACTR201908560004686 and PACTR201504000771349), the Netherlands (NL7718 and EUCTR2018-000283-28-NL), Egypt (NCT03717701, NCT03213639 and NCT03724838) and Iran (IRCT2017082333680N2). A trial of 400 women with severe pre-eclampsia found that a resveratrol and nifedipine combination reduced the time needed to control blood pressure compared to nifedipine alone, but did not improve maternal or neonatal outcomes.(75) It is unclear if the trial was powered to detect changes in maternal or neonatal outcomes.

*Phase II*

Remaining candidates in Phase II were ranked low potential (Curcuma longa extract, serelaxin, sildenafil citrate, tadalafil, Iloprost and ozagrel), due to a lack of clinical data. The safety concerns regarding sildenafil (when used for other indications in pregnancy) is noteworthy,(76) though its use in preventing intrapartum fetal distress is currently being investigated.(77)

*Phase I*

Vardenafil, *Purnica granatum* extract (pomegranate juice), conestat alfa, RMC 035 and S-Nitrosoglutathione all ranked low, due to a lack of available information for most variables; cold-chain transport and storage is also required for *Purnica granatum* extract (pomegranate juice), conestat alfa, S-Nitrosoglutathione and RMC 035 in their current formulations.
